# Pre-hospital CPAP for acute respiratory failure: the ACUTE feasibility and pilot randomised controlled trial

**DOI:** 10.29045/14784726.2019.12.4.3.53

**Published:** 2019-12-01

**Authors:** Gordon W. Fuller, Samuel Keating, Steve Goodacre, Esther Herbert, Gavin Perkins, Andy Rosser, Imogen Gunson, Matthew Ward, Josh Miller, Mike Bradburn, Praveen Thokala, Tim Harris, Maggie Marsh, Alex Scott, Cindy Cooper

**Affiliations:** University of Sheffield; University of Sheffield; University of Sheffield; University of Sheffield; University of Warwick; West Midlands Ambulance Service University NHS Foundation Trust; West Midlands Ambulance Service University NHS Foundation Trust; West Midlands Ambulance Service University NHS Foundation Trust; West Midlands Ambulance Service University NHS Foundation Trust; University of Sheffield; University of Sheffield; Barts and the London School of Medicine and Dentistry; University of Sheffield; University of Sheffield; University of Sheffield

## Abstract

**Introduction:**

Acute respiratory failure (ARF) is a common and life-threatening medical emergency. Continuous positive airway pressure (CPAP) is a potentially beneficial alternative treatment; however, it is uncertain whether this could improve important outcomes in NHS ambulance services. The ACUTE study aimed to assess the feasibility of a large-scale pragmatic trial of pre-hospital CPAP.

**Methods:**

The study was a pilot randomised controlled trial of the O-Two system CPAP mask versus standard oxygen therapy, with concealed allocation in identical sealed boxes. Feasibility objectives estimated the incidence of eligible patients; the proportion recruited and allocated to treatment appropriately; adherence to allocated treatment; and retention and data completeness. The primary clinical endpoint was 30-day mortality. Ancillary studies included an ARF incidence study, ARF diagnostic agreement study, clinician perceptions of CPAP mixed methods study and investigation of allocation concealment.

**Results:**

Over 12 months, 77 patients were enrolled (target 120). CPAP was fully delivered in 74% (target 75%). There were no major protocol violations/non-compliances. Full data were available for all key outcomes (targets ≥ 90%). Thirty-day mortality was 27.3%. Of deceased patients, 14/21 (68%) either did not have a respiratory condition or had ceiling of treatment decisions implemented excluding hospital NIV and critical care.

**Conclusion:**

The ACUTE trial recruitment rate was below the target rate and feasibility was not demonstrated. Identification of patients who might benefit from pre-hospital CPAP was challenging. It appeared difficult to exclude conditions where CPAP would not work, or might be harmful, and to select appropriate patients where there was a meaningful chance of success, or where the potential advantages of pre-hospital CPAP would outweigh the burdens of more advanced and aggressive treatment. The limited compliance with CPAP, and the difficulty in identifying patients who could benefit from CPAP, indicate that pre-hospital CPAP is unlikely to materially reduce mortality. A definitive effectiveness trial of CPAP in the NHS is therefore not recommended.

